# Neuropsychological impact of Sanda training on athlete attention performance

**DOI:** 10.3389/fpsyg.2024.1400835

**Published:** 2024-09-23

**Authors:** Yuzhu Teng, Hailan Wu, Xiaoyun Zhou, Feiyang Li, Zhong Dong, Huafeng Wang, Kai Wang, Qianchun Yu

**Affiliations:** ^1^Department of Maternal, Child and Adolescent Health, School of Public Health, Anhui Medical University, Hefei, Anhui, China; ^2^Department of Social Medicine and Health Service Management, School of Health Management, Anhui Medical University, Hefei, Anhui, China; ^3^Department of Neurology, The First Affiliated Hospital of Anhui Medical University, Hefei, Anhui, China; ^4^Sanda Teaching and Research Office, Wushu College, Beijing Sport University, Beijing, China; ^5^Department of Health Information, School of Health Management, Anhui Medical University, Hefei, Anhui, China; ^6^Sports Human Science Research Center, School of Humanistic Medicine, Anhui Medical University, Hefei, Anhui, China

**Keywords:** Sanda, executive control, attention networks, neuropsychological assessment, cognitive function

## Abstract

**Background:**

Sanda, a martial art that primarily involves punching, kicking, and throwing techniques, requires athletes to maintain high levels of concentration during combat. Sanda principally involves striking the opponent to secure victory, with trauma frequently occurring to the head; however, it remains unclear whether prolonged training enhances or impairs the athletes' attentional capacities. This study aimed to investigate the impact of Sanda training on athletes' attention by employing attention network tests.

**Methods:**

A retrospective analysis was conducted on 37 professional Sanda athletes from a certain sports academy; 38 college students from the same institution majoring in physical education were recruited as the control group. Control participants had no prior experience in Sanda training, and the Sanda and control groups were matched in terms of sex, age, and education level. The Attention Network Test (ANT) was administered to both groups to compare differences in efficiency across the alerting, orienting, and executive control networks.

**Results:**

Compared to the control group, the Sanda athletes exhibited significantly higher executive control network efficiency values and executive control network efficiency ratios (*P* < 0.05). There were no significant differences between the Sanda group and the control group regarding the efficiency values of the alerting and orienting networks (*P* > 0.05). Additionally, total accuracy and total reaction time between the Sanda athletes and control participants showed no statistically significant differences (*P* > 0.05).

**Conclusion:**

Sanda practice has detrimental effects on attention, including a decrease in the efficiency of the executive control network and a prolongation of the total reaction time. Therefore, athletes should improve attention training and use head protection to prevent frequent head impacts during training.

## 1 Introduction

Sanda (Chinese kickboxing) is a hand-to-hand combat sport that utilizes punches, kicks, and wrestling techniques. The rapid and frequent transitions between attack and defense in competition require the athlete to concentrate on accurate and rapid reactions. Attention forms the basis of a sparring athlete's response to an opponent's punches and kicks; it is a preparatory attentional state for an anticipated cognitive or behavioral activity. During combat, the athletes' cognitive systems must respond to various stimuli as quickly as possible to ensure victory. Therefore, good athletes should have a good attention span. Angelini (2008) performed transcranial magnetic simulations on athletes and non-athletes, and found that the athletes responded more strongly when their motor areas were stimulated when compared to controls. However, as the head and face score higher than the trunk in the rules of sparring, the head and face are the most important parts of the body for effective striking, resulting in more injuries to the head (Fang, 2013). Specific attentional network deficits have been reported in people who have suffered mild traumatic brain injuries including concussion symptoms (Wang et al., 2023). The attentional changes in professional Sanda athletes who have undergone long-term training are unclear. Whether attention is improved by prolonged focused training or impaired by common sports injuries is worth investigating further.

In recent years, the association between physical activity and cognition has attracted the interest and attention of researchers in many fields. Attention is an important component of the cognitive processes. Attention was initially viewed as a single system; however, further exploration revealed that simple theoretical models do not account well for the processing of attention. Posner and Petersen (1990) proposed an attention network model based on a large number of cognitive neuroscience studies. This model structurally and functionally divides attention into three subsystems: alerting, orienting, and executive control. These three systems have different brain region localizations and relatively independent neural and biochemical systems; they are regarded as one of the most influential theoretical models of attention (Petersen and Posner, 2012; Posner, 2023; Klein et al., 2024). The alertness network maintains vigilance in preparation for receiving incoming information; it is associated with the frontal and parietal lobes of the right cerebral hemisphere and relies on the norepinephrine system. The orienting network selectively processes incoming information; it focuses on sensory events through spatially shifted attention, which is associated with the temporoparietal junctional area and the parietal lobule and relies on the cholinergic system. The executive control network develops plans, monitors and resolves conflict, and is associated with the anterior cingulate gyrus; it relies on the dopamine system (Davidson and Marrocco, 2000; Coull et al., 2001; Fan et al., 2003).

Fan et al. (2005) designed the Attention Network Test (ANT). It is an intuitive tool that is easy to use and employs both spatial cueing and lateral inhibition. Spatial cueing examines the efficacy of the two loops, the alerting network, and the orienting network, whereas lateral inhibition examines the efficacy of the executive control network. The ANT can assess all three of these networks quantitatively and is now widely used in children with attention deficit hyperactivity disorder, in Alzheimer's populations, and in normal populations (Ishigami et al., 2016; McDonough et al., 2019; Vázquez-Marrufo et al., 2019). All behavioral activities of the human body are governed by the central nervous system, including an athlete's understanding, mastery, and application of difficult techniques. Johnstone and Marí-Beffa (2018) assessed the effects of martial arts training on the alerting network, orienting network, and executive control network using the ANT. Adults with at least 2 years of martial arts training experience were selected as the experimental group for the study, covering a wide range of martial arts styles such as Karate, Taekwondo, Kickboxing, Jujitsu, Tai Chi, Judo, Thai Boxing, and Kung Fu. The results showed that, compared to the control group, the martial arts training group showed a significant improvement in performance on the alerting network. Although their study provides valuable insights, its population of mainly healthy adults with short training periods and the variety of martial arts styles covered may have led to results that are somewhat generalized and lacking in specificity. There is a lack of research related to the cognitive aspects of attentional changes in professional sparring athletes.

Neuropsychological (NP) testing is an objective measure of the brain's behavioral associations and is more sensitive to minor cognitive deficits than clinical examination. Therefore, this study aimed to investigate the impact of a specific martial arts style (Sanda training) on athletes' attention by employing ANTs. Prolonged and intense training may have a different effect on the attentional network than other martial arts styles. It was hypothesized that better attentional performance might be observed in athletes exposed to Sanda compared to controls.

## 2 Materials and methods

### 2.1 Participants

We randomly selected 37 players from the Beijing Sport University Sanda Team, which had won the National Sanda Championship, including 32 (86.5%) males and five (13.5%) females. There were 18 first-string players (15 males and three females) and 19 second-string players (17 males and two females), with an average age of 22.5 ± 1.8 years and 14.9 ± 1.8 years of education. The 37 athletes mentioned above were included in the Sanda group.

During the same period, 38 college students majoring in physical education who had never been exposed to Sanda were randomly selected at Beijing Sport University. There were 32 (84.2%) males and six (15.8%) females with a mean age of 22.1 ± 2.0 years and 15.3 ± 1.5 years of education. The above 38 students were included in the control group. The basic characteristics of all the participants in the study are shown in Table 1. There were no statistically significant differences between the two groups of participants in terms of sex (χ^2^ = 0.078, *P* = 0.781), age (*t* = −1.009, *P* = 0.316), and years of education (*t* = 1.178, *P* = 0.243).

**Table 1:** Basic characteristics of included and excluded participants.

**Basic characteristics**	**Sanda group (*n* = 37)**	**Control group (*n* = 38)**	***P-*value**
Age/years (*M* ± SD)	22.5 ± 1.8	22.1 ± 2.0	0.316
**Sex**			
Male	32/86.5	32/84.2	0.781
Female	5/13.5	6/15.8	
**Ethnicity**			0.513
Han nationality	36/97.3	35/92.1	
Hui nationality	1/2.7	2/5.3	
Other nationalities	0/0	1/2.6	
Education levels/years (*M* ± SD)	14.9 ± 1.8	15.3 ± 1.5	0.243
**Family monthly income per capita/yuan (** ***n*** **/%)**			0.749
< 1,000	1/2.7	0/0.0	0.749
1,000–2,500	7/18.9	8/21.1	
2,501–4,000	15/40.5	17/44.7	
>4,000	14/37.8	13/34.2	
**Place of residence (** ***n*** **/%)**			0.062
Urban areas	33/89.2	35/92.1	
Rural areas	4/10.8	62/7.9	
Smoking (*n*/%)	2/5.4	1/2.6	0.540
Alcohol drinking (*n*/%)	4/10.8	7/18.4	0.352

Participants with any of the following conditions were excluded from the study: history of brain trauma, nervous system or mental illness, or previous concussion. None of the participants we included were excluded. The naked or corrected visual acuity of the participants were normal, and they were all right-handed.

All procedures performed in studies involving human participants were in accordance with the ethical standards of the institutional and/or national research committee and with the 1964 Helsinki Declaration and its later amendments or comparable ethical standards. The study was approved by the Committee of Bio-Medical Ethics of Anhui Medical University (No: 82230093). Informed consent was obtained from all individual participants and legal guardians included in the study.

### 2.2 Study procedures

#### 2.2.1 ANT

**Figure 1:**
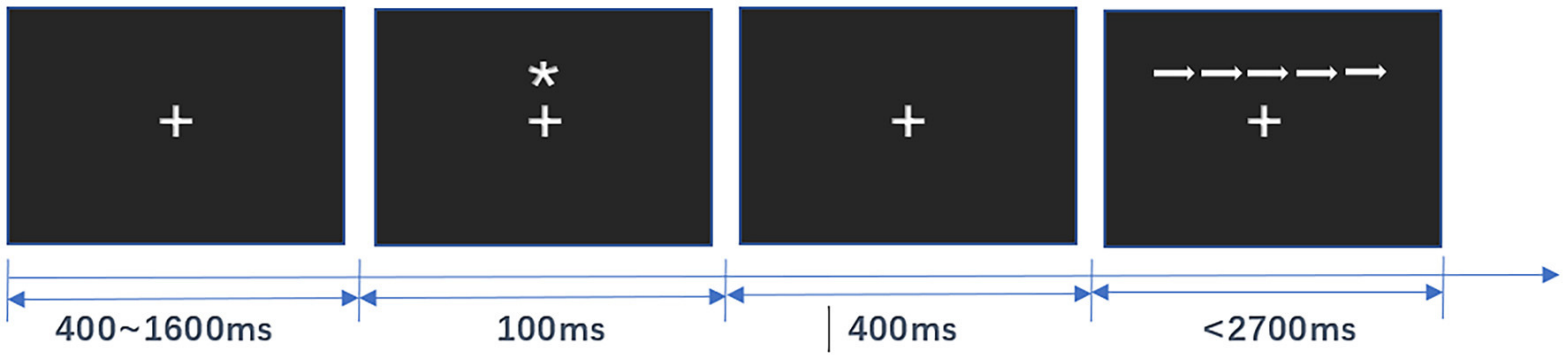
Schematic diagram of the Attention Network Test (ANT) process.

Attentional network function was assessed using the ANT in both the Sanda group and the control group. During the experiment, participants sat at a distance of 40 cm from the center of the computer screen, with their eyes fixed on the gaze point in the center of the screen and their fingers placed on the response keys of the keyboard, and were asked to correctly and quickly determine the direction of the target, i.e., whether the direction of the arrow in the center was to the left or to the right, and to respond by pressing the corresponding key: if the arrow is to the left, press the “←” key; if the arrow is to the right, press the “ → ” key. For each trial, the center gaze point “+” was first presented for 400–1,600 ms, followed by the cue “^*^” for 100 ms. The center gaze point was presented again for 400 ms alone, and finally, the target stimulus was presented in the center of the screen for no more than 2,700 ms (Figure 1). The target stimulus disappeared as soon as the participant responded by pressing a key.

**Figure 2:**
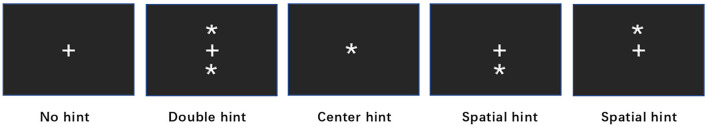
Different hint states in the Attention Network Test (ANT).

**Figure 3:**
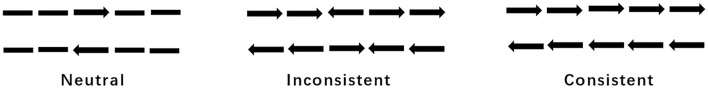
Different target stimulus types in the Attention Network Test (ANT).

The gaze point was presented in the center of the screen throughout the study. There were four types of cues (Figure 2): (1) no hint: only the center gaze point was present without hints; (2) center hint: a single hint was presented at the center gaze point; (3) double hint: the two hints were presented above and below the center gaze point; and (4) spatial hint: a single hint was presented above or below the center gaze point. No hint, center hint, and double hint could not predict the upcoming location of the target stimulus, whereas spatial hints could predict the location of the target stimulus (the target stimulus appeared at the spatial cue presentation). The target stimulus was a left- or right-facing arrow, flanked by two-line segments that were the same length as the target stimulus, with or without arrows. The target stimuli were categorized into three situations (Figure 3): (1) neutral: no arrows on either side of the line segments; (2) consistent: arrows on both sides of the line segments, pointing in the same direction as the target stimulus; and (3) inconsistent: arrows on both sides of the line segments, pointing in the direction opposite to the direction of the target stimulus.

The experiment consisted of 312 formal trials and 24 practice sessions. The participants were familiarized with the targets and interference terms involved in this study through feedback on both correct and incorrect results. The entire experiment lasted approximately 30 min and was divided into three phases, with subjects allowed to rest between each phase. Since the participants were affected by factors such as interference with hints and target states during the experiment, the correct and incorrect responses and times of the participants were recorded in real time, and the attentional network efficacy was assessed by determining the response time in different states.

#### 2.2.2 Efficiency calculation for attention network

According to the ANT principle devised by Fan et al. (2002), attentional network efficiency can be calculated as follows: alerting network efficiency = response time (RT) no hint condition – RT double hint condition. This is because attention is more dispersed in the no hint condition, and the appearance of a double hint asterisk at the location where the target is likely to appear will cause attention to be focused on the location where the target will appear, which will result in a reduction in the RT (alerting effect); larger values suggests a stronger alerting effect. The orienting network efficiency was calculated as follows: orienting network efficiency = RT center hint condition – RT effective spatial hint condition. This is because the effective spatial hint will provide information about localization, i.e., the location where the target will appear, which has an orienting effect and will shorten the participant's RT; larger values suggest a higher efficiency of the orienting network. Executive control network efficiency was calculated as follows: control network efficiency = RT direction inconsistent target stimulus condition – RT direction consistent target stimulus condition. When the direction of the target arrow is inconsistent with the surrounding arrows, the attentional network needs to resolve this conflict, extending the RT compared to the direction-consistent condition. Therefore, a small value suggests a strong executive control function, which is different from the first two network indicators. To exclude the possible effect of differences in total ANT reaction times, the efficiency ratio for each network was calculated separately as follows: efficiency ratio for each network = the efficiency value for that network (i.e., the difference in reaction times)/total reaction time. To confirm that the four cue types and three target stimulus conditions were appropriately manipulated in the experiment, the RT was collected for each cue type and target stimulus condition, and the mean RT and standard deviation were calculated. Differences in RTs across cue types and target stimulus conditions were compared.

### 2.3 Statistical analysis

Continuous variables are described as means and standard deviations, and categorical variables as numbers (percentages). The effects of cue type and target stimulus condition on RT were examined using the one-way ANOVA with *post hoc* tests using the Bonferroni method. The independent sample *t*-test was used to compare the ANT scores of the Sanda group and control group. Main effect sizes (ES) were presented as Hedges' *g* for *t*-test calculations. For the simple effect analysis, Hedges' *g* was used as the ES for small sample sizes with ranges of 0.2–0.6, 0.61–1.19, and 1.2, representing small, medium, and large effects (Hopkins et al., 2009). Generalized linear models were adopted to understand the association of Sanda sports with the athletes' attention network. Confounding factors were adjusted for in the models to test the robustness of the findings. Statistical Product and Service Solutions version 26.0 (Armonk, NY, USA) was utilized for data analyses.

## 3 Results

### 3.1 Effects of different cue types and target stimulus conditions on RT

There was a significant difference in the RT between the four cue types [*F* = 121.14, *P* < 0.001, partial eta squared ($\eta_{\text{p}}^{2}$) = 0.021; Table 2]. The *post hoc* testing suggested that the RT with no hint > RT with a center hint > RT with a double hint > RT with a spatial hint. It also showed a significant difference in the RT among the three target stimulus conditions (*F* = 1263.06, *P* < 0.001, η^2^_*p*_ = 0.128), with *post hoc* testing suggesting that the RT to the incongruent stimuli > RT to the congruent stimuli > RT to the neutral stimuli.

**Table 2:** Effects of different cue types and target stimulus conditions on reaction time.

	**Reaction time (ms; mean ±SD)**	***F*-values**	***P*-values**	**η^2^*_*p*_***
**Types of cues**		121.14	< 0.001	0.021
No hint	652.9 ± 188.6			
Center hint	621.7 ± 177.8			
Double hint	608.3 ± 173.4			
Spatial hint	580.0 ± 178.2			
**Situations of target stimuli**		1,263.06	< 0.001	0.128
Neutral	544.2 ± 167.6			
Consistent	601.5 ± 162.9			
Inconsistent	701.3 ± 177.4			

### 3.2 Comparison of ANT between the Sanda and control groups

**Figure 4:**
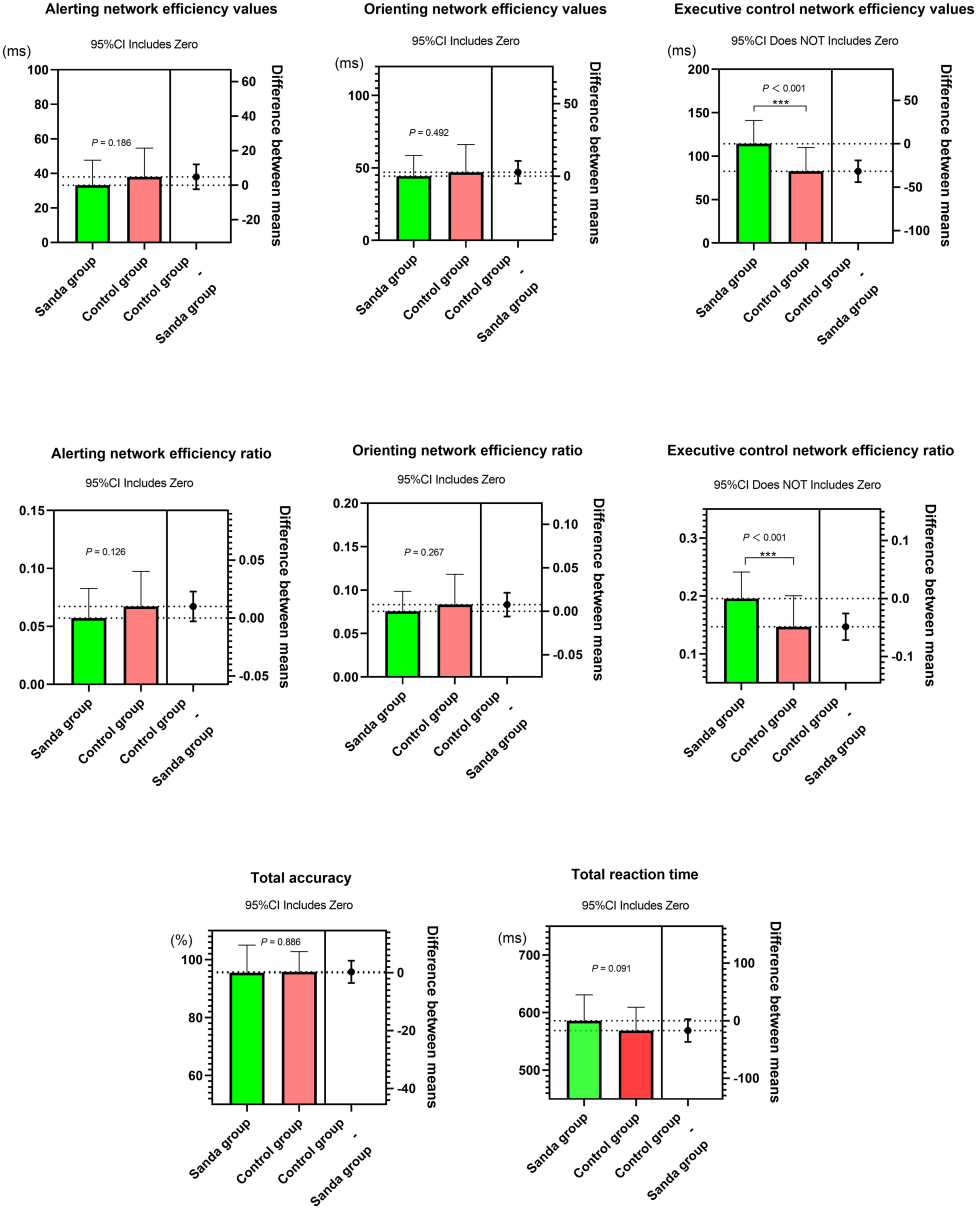
Comparison of the Attention Network Test (ANT) results between the Sanda group and control group. The *Y*-axis on the left represents the original data of the two groups, and the *Y*-axis on the right shows the effect estimation (group mean difference and its 95% confidence interval). ^***^p < 0.001.

As shown in Figure 4, the results of the independent sample *t*-test showed that there was no significant difference between the alerting network efficiency [33.2 ± 14.5 vs. 38.0 ± 16.7, Hedges'g = 0.30, 95% confidence interval (CI): −0.15 to 0.76], orienting network efficiency (44.4 ± 14.3 vs. 47.1 ± 19.1, Hedges' *g* = 0.16, 95% CI: −0.29 to 0.61), total RT (585.8 ± 45.0 vs. 568.9 ± 40.6, Hedges' *g* = 0.40, 95% CI: −0.06 to 0.85), and total accuracy (95.5 ± 9.5 vs. 95.8 ± 7.0, Hedges' *g* = 0.03, 95% CI: −0.42 to 0.49) in the Sanda group compared to the control group (all *P* > 0.05). The executive control network efficiency values were higher in the Sanda group than in the control group (114.2 ± 26.8 vs. 82.6 ± 27.3, Hedges' *g* = −1.17, 95% CI: −1.66 to −0.68, *P* < 0.05). Excluding the effect of the total RT, the executive control network efficiency ratio of the Sanda group was still higher than that of the control group (0.20 ± 0.05 vs. 0.15 ± 0.05, Hedges' *g* = −0.98, 95% CI: −1.46 to −0.5, *P* < 0.05). The alerting network efficiency ratio (0.06 ± 0.03 vs. 0.07 ± 0.03, Hedges' *g* = 0.36, 95% CI: −0.10 to 0.81) and the orienting network efficiency ratio (0.08 ± 0.02 vs. 0.08 ± 0.03, Hedges' *g* = 0.26, 95% CI: −0.20 to 0.71) in the Sanda group were not significantly different from those in the control group (both *P* > 0.05).

### 3.3 Association between Sanda and attentional function

As shown in Table 3, without adjusting for any confounders (Model 1), the executive control network efficiency value was 31.66 (95% CI: 19.57–43.76) ms higher in the Sanda group compared to the control group, suggesting a decrease in executive control function. Excluding the effect of the total RT, the executive control network efficiency ratio was higher in the Sanda group compared to the control group (β = 0.05, 95% CI: 0.03–0.07). After adjusting for confounders (Model 2), the executive control network efficiency value was 34.11 (95% CI: 22.40–45.83) ms higher in the Sanda group compared to the control group. Higher executive control network efficiency ratios were observed in the Sanda group compared to the control group (β = 0.05, 95% CI: 0.03–0.08).

**Table 3:** Generalized linear model for the association between Sanda and athletes' attention networks (β and 95% CI).

**ANT**	**Sanda sports exposure**	
	**Model 1**	**Model 2**
Alerting network efficiency values	−4.81 (−11.79 to 2.17)	−3.94 (−10.57 to 2.69)
Orienting network efficiency values	−2.70 (−10.26 to 4.86)	−3.38 (−10.94 to 4.18)
Executive control network efficiency values	31.66 (19.57 to 43.76)^***^	34.11 (22.40 to 45.83)^***^
Alerting network efficiency ratio	−0.01 (−0.02 to 0.00)	−0.01 (−0.02 to 0.00)
Orienting network efficiency ratio	−0.01 (−0.02 to 0.01)	−0.01 (−0.02 to 0.01)
Executive control network efficiency ratio	0.05 (0.03 to 0.07)^***^	0.05 (0.03 to 0.08)^***^
Total reaction time	16.92 (−2.20 to 36.03)	16.24 (−2.80 to 35.28)
Total accuracy	−0.28 (−4.01 to 3.45)	−1.00 (−4.39 to 2.38)

ANT, attention network test.

Model 1: unadjusted model.

Model 2: adjusted for age, sex, ethnicity, education levels, family monthly income per capita, place of residence, smoking and alcohol drinking history.

^***^P < 0.001.

## 4 Discussion

This study revealed that exposure to the sport of Sanda may adversely affect the athletes' attentional network, as evidenced by elevated values of the executive control network efficiency, suggesting a decrease in executive control functioning. After excluding the effect of the total RT, the efficiency ratio of the executive control network remained higher in the Sanda group, indicating the stability of the results.

Attention is the process of pointing and focusing mental activity and consciousness on certain information or objects, in addition to the complex process of the appropriate allocation and processing of relevant sensory stimuli by the brain, which is a cognitive process. Whether exposure to Sanda affects an athlete's attention network remains controversial. Sanda requires focused attention during sparring, including scanning the opponent's body for scoring points, which requires the involvement of the alert network. Avoiding and blocking any incoming hit from the opponent requires orienting network efficiency for Sanda athletes. Furthermore, when the opponent uses feigned punches and kicks to distract attention, it is necessary to implement an executive control network to address the conflict. Johnstone and Marí-Beffa (2018) found that practitioners have a higher efficiency in their alerting networks than the control group when exploring the impact of extensive training in martial arts on cognitive control in adults. However, we observed no significant effect of long-term Sanda training on the alerting network. The reasons for the differences in these findings may lie in the differences in the martial arts styles, in addition to differences in the training intensity and years of training. A variety of martial arts styles were involved in the study by Johnstone and Marí-Beffa's (2018), of which Jujitsu, Tai Chi, and Judo typically do not involve or prohibit head strikes. In contrast, our study focused on Sanda athletes with frequent head strikes, which may have different effects on cognitive functioning. Additionally, the participants in the Johnstone and Marí-Beffa (2018) study were healthy adults with 2 years of training experience, whereas our study focused on professional Sanda athletes who had undergone more intense training over a longer period. Long-term, high-intensity training may lead to different changes in cognitive functioning in professional athletes than in short-term trainers. Although our study did not find a significant difference between the Sanda athletes and the control group in terms of the vigilance network efficiency, the mean values of the alerting network efficiency were lower in the Sanda athletes than in the control group (*P* > 0.05). Our findings suggest that the potential damage associated with head blows in professional athletes under prolonged, high-intensity training may outweigh the improvement in their alertness from training. The influence of these factors needs to be explored in depth in further studies. Furthermore, Sanda is a contact sport where the practice of striking the head to score points exposes athletes to the cumulative effects of non-severe head injuries, such as sports concussions (Prien et al., 2018). Howell et al. (2013) found that concussion resulted in prolonged RTs on laboratory tests of attention and executive function for up to 2 months post-injury. Our study suggests that prolonged exposure to Sanda may cause a decrease in executive control functions and that Sanda athletes do not show a significant decrease in performance concerning the alerting network, orienting network, and RT.

The inconsistency in the findings could be attributed to the differences in the methods, sample sizes (Noordzij et al., 2010), and instruments used for assessing the athlete's neuropsychological development (Merritt et al., 2017). Interceptive sports, such as boxing, Sanda, table tennis, and archery, which require a high degree of body coordination or precise maneuvering with an instrument (e.g., bat or sword), have different effects on attentional regulation. In particular, an exploratory study has shown that archery can enhance attentional regulation (Diotaiuti et al., 2021; Lu et al., 2021). In contrast, our previous study found that boxing may negatively affect cognitive function (Teng et al., 2022). Combined with the results of the current study, it is hypothesized that the effect of Sanda on attention may depend primarily on whether the athlete suffers a blow to the head, in addition to the frequency and intensity of the blow.

The mechanisms underlying the association between long-term exposure to sporadic fighting and declines in executive control function in athletes are unclear. Executive control function is closely related to the neural circuits regulated by the cingulate gyrus (Uddin, 2021). Neuroimaging techniques have shown that prolonged training in motor skills leads to a plastic reorganization of brain structure and function in professional athletes engaged in a variety of sports (Huang et al., 2015). A longitudinal study with a follow-up period of up to 1 year found that the cingulate cortex of combatants was significantly thinner than at baseline, which was accompanied by a decrease in N-acetyl aspartate metabolites (Mayer et al., 2015). Additionally, animal model studies have shown that repeated head blows result in greater neuropathological and neurobehavioral changes than a single head blow (Kane et al., 2012). Consequently, prolonged performance of combat sports, such as boxing practice, may affect the normal regulation of executive control functions by altering brain structure and function, in addition to adjusting the signaling molecules related to neurometabolism.

The strengths of this research are as follows. (1) The present study provides preliminary evidence on the effects of Sanda on attentional networks, with a particular focus on changes in the efficiency of executive control networks, filling a research gap on cognitive impacts in sports, particularly in the field of Sanda. (2) This study retrospectively analyzed professional Sanda athletes and compared them to a control group that had not been exposed to Sanda training. The controlled validity of the study design increases the likelihood of finding a causal association.

There are also some limitations to this study. First, this study had a retrospective design and cannot provide direct evidence of causality. Although athletes and controls were matched, some confounding factors could not be controlled for. Second, the frequency, intensity, and protection of blows to the head in the Sanda athletes were not recorded in detail, which may affect the precise interpretation of changes in attention.

## 5 Conclusion

In conclusion, the correct interpretation of neuropsychological test results can provide a basis for assessing changes in cognitive functioning in athletes during exercise. Therefore, this study suggests that neuropsychological testing can be used regularly in professional athletes to help identify and prevent early brain injuries that are difficult to recognize with instruments, in addition to the long-term complications.

## Data Availability

The raw data supporting the conclusions of this article will be made available by the authors, without undue reservation.
